# Molecular Characteristics of the Fatty-Acid-Binding Protein (FABP) Family in *Spirometra mansoni*―A Neglected Medical Tapeworm

**DOI:** 10.3390/ani13182855

**Published:** 2023-09-08

**Authors:** Shinan Liu, Fei Gao, Ruijie Wang, Wen Li, Siyao Wang, Xi Zhang

**Affiliations:** Department of Parasitology, School of Basic Medical Sciences, Zhengzhou University, Zhengzhou 450001, China; liushinan940914@163.com (S.L.); gaofei199812@163.com (F.G.); 17839975524@163.com (R.W.); liwen01232022@163.com (W.L.); wangsiyao2021@126.com (S.W.)

**Keywords:** tapeworm, *Spirometra mansoni*, fatty-acid-binding protein, gene expression, molecular characterization

## Abstract

**Simple Summary:**

The purpose of this study was to investigate the members, gene expression patterns, and molecular characteristics of the *Spirometra mansoni* fatty-acid-binding protein family. We screened and characterized 11 new SmFABP genes and divided them into two groups based on conservative motifs. All 11 genes were highly expressed in the adult stage. We found that SmFABPs had a high genetic diversity and the FABPs in medical platyhelminthes were relatively conserved. When rSmFABP is combined with palmitic acid, a more stable holographic morphology can be established to protect the hydrolysis site of FABP.

**Abstract:**

The plerocercoid larva of the tapeworm *Spirometra mansoni* can parasitize humans and animals, causing serious parasitic zoonosis. The molecular characteristics and adaptive parasitism mechanism of *Spirometra* tapeworms are still unknown. In this study, 11 new members of the fatty-acid-binding protein (FABP) family were characterized in *S. mansoni*. A clustering analysis showed 11 SmFABPs arranged into two groups, and motif patterns within each group had similar organizations. RT–qPCR showed that SmFABPs were highly expressed in the adult stage, especially in gravid proglottid. A high genetic diversity of SmFABPs and relative conservation of FABPs in medical platyhelminthes were observed in the phylogenetic analysis. Immunolocalization revealed that natural SmFABP is mainly located in the tegument and parenchymal tissue of the plerocercoid and the uterus, genital pores, and cortex of adult worms. rSmFABP can build a more stable holo form when binding with palmitic acid to protect the hydrolytic sites of the protein. A fatty acid starvation induction test suggested that SmFABP might be involved in fatty acid absorption, transport, and metabolism in *S. mansoni*. The findings in this study will lay the foundation to better explore the underlying mechanisms of FABPs involved in *Spirometra* tapeworms as well as related taxa.

## 1. Introduction

*Spirometra mansoni* (Cestoda: Diphyllobothriidea) is a neglected human infective tapeworm. Its motile larva (plerocercoid) can lodge in the subcutaneous tissues and sometimes invade the abdominal cavity, eyes, and brains of humans, causing a parasitic zoonosis known as sparganosis [1]. Humans are mainly infected by eating raw or undercooked infected intermediate hosts (mainly frogs and snakes), by drinking water contaminated with copepods that have been infected with procercoids, or by placing frog or snake flesh on open wounds [2,3]. More than 2000 human sparganosis cases have been reported thus far, with the majority coming from Eastern and Southeastern Asia [4,5,6]. Less frequently, human cases have also been reported in Africa, America, and Europe [7,8]. 

Complicated mechanisms and interactions of plerocercoids with the host during infection are the causes of serious pathological changes that lead to a fatal outcome. Understanding the intricacies of the parasite and its interactions with hosts is central to developing intervention strategies [9]. Several molecules have been implicated in interference with the host response, such as fatty-acid-binding proteins (FABPs) [4,10,11]. FABPs have specific patterns of tissue distribution and are associated with a wide range of crucial biological processes, including the regulation of cellular lipid homeostasis, cell growth and differentiation, cellular signaling, gene transcription, and the immune response [12,13,14]. Helminth parasites that live in an oxygen-deprived environment in the gastrointestinal tract of their hosts are unable to synthesize most of their own lipids de novo, in particular long-chain fatty acids and cholesterol [15]. FABPs are important to those helminths for the biosynthesis of fatty acids and cholesterols because they depend on fatty acids from the host for intracellular lipid oxidation via transportation by FABPs [16]. Owing to their important role in lipid oxidation, FABPs are attractive as targets of new anthelminthic drugs or vaccines [17]. Since the first FABP was identified in the intestinal mucosa [18], fatty-acid-binding proteins have been identified in various species of nematodes, trematodes, and cestodes [16,19,20,21,22]. Furthermore, a comparative genomic analysis showed that the FABP family is highly expressed in *S. mansoni* [23,24], indicating the crucial roles of FABPs in *S. mansoni*. Nevertheless, FABPs have not been studied in *Spirometra* species thus far, and we know nearly nothing about the family structures and molecular features of FABPs in this medical tapeworm.

FABPs constitute a multigenic family of small cytosolic proteins (~15 kDa) that mainly noncovalently bind hydrophobic ligands of fatty acids [25]. During long-term evolution, multiple gene duplications have occurred in this gene family, giving rise to 12 FABPs in vertebrates, and more than 30 FABP genes have been found in a wide range of invertebrates [13,26,27]. Despite the variable sequence identity of different FABPs, all these proteins share a conserved structure composed of ten antiparallel beta-strands and two alpha-helixes [28]. Previous studies have suggested that FABPs evolved via successive gene duplications, generating a large number of tissue-specific homologues [29,30]. However, systematic and genome-wide investigations of FABPs in platyhelminthes remain in their infancy. With an increasing number of genomic data for platyhelminth parasites, it is feasible to explore their genomic organization and gene structures and phylogenetic patterns of FABP genes over a larger scope. More specifically, the following objectives were addressed in this study: to investigate protein family members, gene expression, and molecular characterizations of FABP in *S. mansoni*, as well as to explore the gene organization and phylogenetic patterns of FABPs across parasitic platyhelminthes.

## 2. Materials and Methods

### 2.1. Ethical Approval

The study was conducted according to the guidelines of the Declaration of Helsinki, and all procedures involving animals were approved by the Life Science Ethics Committee of Zhengzhou University (Permit code. SYXK 2020-0821). The animals were handled in accordance with good animal practices required by the Animal Ethics Procedures and Guidelines of the People’s Republic of China.

### 2.2. Parasites and Experimental Animals

The plerocercoids of *Spirometra* tapeworms were initially collected from infected frogs (*Pelophylax nigromaculatus*). The collected worms were tentatively identified as *S. mansoni* by molecular typing using the method described in [1]. To obtain adult worms, a single plerocercoid was perorally administered to a 6-month-old specific pathogen free domestic cat. More specifically, the cat was treated with praziquantel at a dose of 5 mg/kg body weight and its feces were checked for 2 weeks. Then, a single plerocercoid was orally fed to the cat. After that, the feces of the infected cat were examined daily. After 40 days from the infection, the cat was pre-anaesthetized then euthanized and the adult worm recovered from the small intestine. The collected adult worm was fixed under a glass plate with AFA (85% Alcohol + 10% Formalin + 5% Glycerol) overnight after washing 3 times with normal saline. The immature, mature, and gravid proglottids were collected and stored at −80 °C until use.

### 2.3. Identification of FABP Family Members in S. mansoni

Genes encoding proteins that contain the fatty-acid-binding protein domain in *S. mansoni* were searched using two databases: (1) the WormBase ParaSite database [31,32] and (2) our transcriptomic data [24]. The extracted sequences were identified as belonging to the fatty-acid-binding protein family by querying for genes annotated with the Pfam domain accession number PF00061. All identified candidates were analyzed using the HMMER tool (https://www.ebi.ac.uk/Tools/hmmer/, accessed on 1 September 2022) to confirm the presence of FABP domains in their protein structure. For candidates from transcriptomic data, the nucleotide sequences were first translated to amino acid sequences, and then the corresponding coding DNA sequences were generated using the NCBI ORF finder tool. Next, the presence of FABP domains in these sequences was confirmed using BLASTX for homology searches. In addition, all retrieved sequences were corroborated by cloning and sequencing of *S. mansoni* cDNAs. The ExPASy server was used to predict the molecular weights, theoretical pI values, and the number of amino acids for all identified SmFABPs. A conserved protein motif analysis was performed using the mixture model via the expectation maximization (MEME) method. The Gene Structure Display Server (GSDS) v2.0 was used to obtain gene features. The ligand-binding residues and domain architecture were identified and documented using the NCBI-CDD server [33]. PSIPRED was used to predict the secondary structure, and the three-dimensional structure was determined using SwissModel. The quality of the 3D model was examined using Ramachandran plot analysis in PROCHECK [34]. In the clustering analysis, multiple sequence alignments were performed in DNAMAN v9.0 software (Lynnon BioSoft, Quebec, QC, Canada). The phylogenetic tree was inferred with the Bayesian inference (BI) method using MrBayes v.3.2 [35] with 10,000,000 generations of sampling trees every 100 generations.

### 2.4. Quantitative RT–PCR Analysis

Quantitative RT–PCR (qRT–PCR) analysis was performed to monitor the expression levels of SmFABPs in the plerocercoid stage as well as different segments of the adult (immature proglottide, mature proglottide, and gravid proglottide). The primers used for qRT–PCR were designed by using the software Primer Premier 5.0 (Premier Biosoft International, Palo Alto, CA, USA). The specific primers are listed in Supplementary Table S1. qRT–PCR was conducted on a 7500 Fast Real-time PCR system (Applied Biosystem, Monza, Italy) with a reaction mixture containing 10 µL of 2 × TB Green Premix Ex Taq (Takara, Japan), 10 μM each of sense and antisense primers, 100 ng of cDNA, and 6.8 μL of ddH_2_O. The real-time PCR program was 95 °C/30 s, then 40 cycles as follows: 3 s at 95 °C and 30 s at 60 °C. GAPDH (GenBank No. AB031067) served as the internal control [9]. Relative expression was calculated using the ΔΔCT method [36].

### 2.5. Sequence Retrieval from Other Platyhelminthes

The FABP sequences of 14 cestodes and 18 trematodes were extracted (Supplementary Table S2). In brief, FABPs were obtained from the WormBase ParaSite database and NCBI using the key words “fatty acid binding protein” based on the following standards: all candidate sequences with an amino acid length between 80 and 180 aa (130 ± 50 aa) were selected for secondary structure prediction to detect if these sequences contain typical structural elements of FABP, and only those with putatively typical structures were regarded as FABP genes [29]. The FABP protein sequence of each species has a genome-wide e-value threshold of 1E-1 and an open and extended gap penalty of 14 and 2, respectively. The turbellarian worm of *Macrostomum lignano* was used as the outgroup in the phylogenetic analysis.

### 2.6. Phylogenetic Analysis

Phylogenetic analyses were performed using two methods of Bayesian inference (BI) and maximum likelihood (ML), respectively. Protein sequences were aligned with MAFFT v7 using the FFT-NS-I method. The best substitution model was defined with the Smart Model Selection (SMS) tool [37] incorporated in PhymL v3.0 [38]. The BI tree was generated with BEAST v1.8.4 [39] using two independent runs of 100,000,000 chains and sampling at every 5000 generations. The software TRACER v1.6 was used to check the convergence of Monte Carlo Markov chains (MCMC) and to ensure adequate effective sample sizes (ESS > 200) after the first 20% of generations were deleted as burn-in. As a result, tree topologies generated by both BI and ML methods were consistent. 

### 2.7. Cloning and Expression of SmFABP

Total RNA was extracted using TRIzol (Invitrogen, Carlsbad, CA, USA). The full-length cDNA sequence of the SmFABP gene (GeneBank No: ON93396) was amplified using specific primers carrying the restriction enzyme sites BamHI and PstI (forward primer: 5′-AAGGATCCATGGAAGCCTTCTGTGGATCA-3′; reverse primer: 5′-AACTGCAGTCAAATTCGGGTGTAGCGT-3′). The PCR products of SmFABP were purified, digested, and cloned into the pMAL-c2X vector (New England Biolabs, Bergen, NJ, USA). The recombinant plasmid was then transformed into *Escherichia coli* BL21 (DE3) (Novagen, La Jolla, CA, USA) and induced by adding IPTG. The optimal expression of SmFABP was obtained at 35 °C under IPTG concentration of 0.5 mM with inducing for 6 h. Then, the bacterial solution containing recombinant protein was crushed using ultrasound at 4 °C for 3 h and purified on an MBP tag protein purification column (Sangon Biotech Co., Shanghai, China). Protein fractions were identified by SDS–PAGE. Images of gels were recorded using ImageScanner (GE Healthcare, Chicago, IL, USA). The purified rSmFABP protein was used to immunize 25 female BABL/c mice (the mice were purchased from Henan Experimental Animal Center) aged 4~6 weeks to prepare anti-rSmFABP immune serum. Antibody titrations of immunized mice were performed by indirect ELISA. In addition, reverse transcription PCR and qRT–PCR were performed to detect the transcriptional levels of the SmFABP gene in different life cycles of *S. mansoni*. Immunofluorescence analysis was performed to determine the expression and localization of SmFABP in different life cycles of *S. mansoni*. Fresh worm bodies were collected and sliced with a thickness of 4 μm. The tissue sections were first retrieved after microwaving for 20 min with a 0.01 M citric acid buffer (pH 6.0), blocking with 5% normal goat serum in PBS, and then incubating at 37 °C for 1 h with 1:100 dilutions of anti-rSmFABP serum, serum of mice infected with plerocercoids, normal mouse serum, serum of the infected cat, and normal cat serum. After washing three times in PBS, the sections were incubated with a 1:50 dilution of Alexa Flour 488-labelled anti-mouse IgG, and the nuclei were stained with 4′,6-diamidino-2-phenylindole (DAPI) at 37 °C for 10 min. Finally, the sections were examined under a fluorescence microscope (Olympus, Tokyo, Japan).

### 2.8. Fatty Acid Binding Assay

To test the fatty acid binding capacity of rSmFABP, we used an SDS–PAGE analysis to determine the proteolytic pattern of rSmFABP binding to fatty acids. The binding of recombinant protein rSmFABP was tested using palmitic acid (C16:0) and oleic acid (C18:1). To obtain the holo forms, 2 μg of purified recombinant SmFABP protein was preincubated for 30 min at room temperature with each fatty acid at a ratio of 1:4 in buffer containing 50 mM Tris–HCl, 50 mM DTT, 2 mM EDTA, and 5 mM CaCl_2_. Then, 1 μg/mL clostripain (ArgC) was added to each reaction and incubated further at 37 °C [16]. The clostripain enzyme, which can hydrolyze the polypeptide chain of protein at the C-terminal end of arginine residues, was used to distinguish the conformations of SmFABP between holo and apo forms [40]. The reactions were collected at 1 h and 18 h after incubation. Then, the reactions were subjected to analysis by SDS–PAGE. Bovine serum albumin (BSA) was added as a positive control. ArgC was not added to negative control reactions.

### 2.9. Fatty Acid Starvation Induction Test

To investigate whether SmFABP participates in the lipid metabolism of plerocercoids, we conducted a starvation treatment on plerocercoids to detect the expression of SmFABP when the supply of exogenous fatty acids was cut off. The plerocercoids were cultured in vitro to test the changes in FABP gene expression under the condition of no fatty acids supplied from the host. In brief, 5 plerocercoids were cultured in RPMI-1640 medium for 0, 6, 12, 18, and 24 h at 37 °C in 5% CO_2_. After incubation, 2 cm was taken from each plerocercoid for RNA extraction. Immediately, all samples were stored in TRIzol (Invitrogen, Carlsbad, CA, USA) at −80 °C until use. RNA of the corresponding worm body was extracted and reverse transcribed into cDNA, and specific primers were designed (F: GGAAGGCAAGACGCTGAAAC; R: CACAAACCAGCTCCTCCAGT) for real-time PCR. The real-time PCR program was 95 °C/30 s, then 40 cycles as follows: 3 s at 95 °C and 30 s at 60 °C. The expression level of FABP at 0 h was used as a control. The transcription of FABP was tested.

### 2.10. Statistic Analysis

All statistical analyses were performed in SPSS for Windows, version 20.0 (SPSS Inc., Chicago, IL, USA). A chi-square test and one-way analysis of variance were used to determine the difference among the groups at various periods. The statistical significance was defined as *p* < 0.05.

## 3. Results

### 3.1. FABP Protein Family in S. mansoni

**Figure 1:**
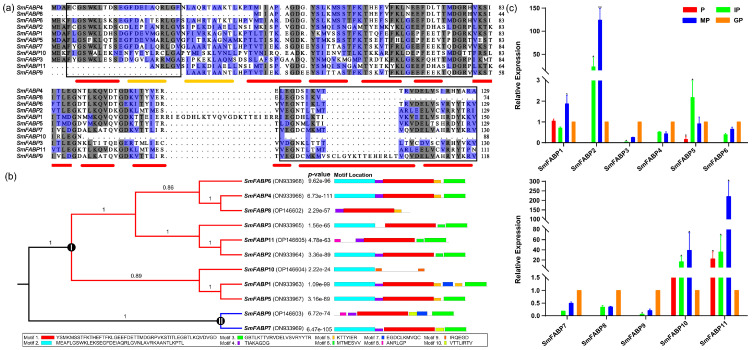
Fatty-acid-binding protein family members identified in *Spirometra mansoni*. (**a**) Multiple alignment of protein sequences of SmFABPs. Three areas are highlighted by black lines, representing the three elements that make up the PRINPS pattern PR00178 (fatty acid binding protein signature), which represents a fingerprint of the FABP family of fatty acids. Amino acids shaded in grey represent conserved positions, while those shaded in purple represent similar positions of amino acids in different proteins. Red bars indicate approximate positions of β-strands (βI-βX), while yellow bars indicate α-helices (αI and αII). They are all based on the EgFABP1 structure (PDB, 1o8v). Secondary structure predictions were performed using the PSIPRED tool. (**b**) Phylogenetic tree and conserved motifs of 11 SmFABPs. (**c**) Quantitative real-time PCR (qRT–PCR) was used to detect SmFABP gene expression in different tissues. GAPDH was used as an internal reference, and 2^−ΔΔCT^ values were used for calculation. All data are presented as the means and standard deviation (SD) with at least three repeats. * indicate significant differences (*p* < 0.01), ** indicate significant differences (*p* < 0.05). P., plerocercoid; IP, immature proglottide; MP, mature proglottide; GP, gravid proglottide.

Initially, 26 sequences containing the FABP domain were identified; however, 15 of them could not be amplified in PCR verification. Therefore, 11 SmFABP sequences were included in the subsequent analyses. These genes were named SmFABP1 through SmFABP11 based on their annotated gene IDs (Table 1). The predicted protein length ranged from 75 aa to 153 aa. The domain length ranged from 69 aa to 148 aa. The molecular weights varied from 8.4 kDa to 17.5 kDa, while the theoretical isoelectric points ranged from 5.00 to 8.09. These predicted sequences were assigned to the FABP family according to three conserved elements found in the PRINTS pattern (fatty-acid-binding protein signature) in their primary structure. The first element consists of the first β-strand (βI) and the first α-helix (αI). The GXW triplet, which is shared by the members of the calycin superfamily, was found in motif 1. The second element contains the end of βIV and the whole βV, and the last includes βIX and βX. These three elements were conserved across all 11 FABPs. The secondary structure showed ten β-strands and two α-helixes located between βI and βII (Figure 1a). A clustering analysis showed 11 SmFABPs arranged into two groups (Figure 1b). Group I has nine SmFABPs, while Group II has only two members (SmFABP7 and SmFABP9). The MEME program identified 10 specific putative motifs containing 6 to 50 residues (Figure 1b). The motif patterns within Group I have similar organizations. For example, motif 2 + motif 4 + motif 1 is the most frequently appearing motif combination in Group I. The 3D homology analysis showed that the protein contains a FABP domain: an intracellular fatty-acid-binding protein domain (residues 3–129) (Supplementary Figure S1). In detail, 27 residues compose the conserved ligand-binding cavity on domain FABP: F4, L16, I19, A20, L23, L29, K30, A33, S38, M40, I42, Y49, M51, I53, Y60, M62, Y64, H72, L91, Q93, M103, S105, L112, M114, A116, R125, and Y127. The motif scan analysis revealed that SmFABP consists of a casein kinase II phosphorylation site (105–108), a cAMP- and cGMP-dependent protein kinase phosphorylation site (125–128), and three protein kinase C phosphorylation sites (7–9, 90–92, 115–117). To profile the expression patterns of SmFABPs, we sampled plerocercoids and different proglottides of adults (immature proglottides, mature proglottides, and gravid proglottides) for analysis by qRT–PCR (Figure 1c). In detail, a total of 11 SmFABPs were found to be expressed in both the plerocercoid and adult stages. All 11 genes were highly expressed in the adult stage, among which 1 was highly expressed in immature proglottids (SmFABP6, ON933968), 4 were highly expressed in mature proglottids, and 6 were highly expressed in gravid proglottids.

### 3.2. Phylogenetic Pattern of FABPs in Parasitic Platyhelminthes

**Figure 2:**
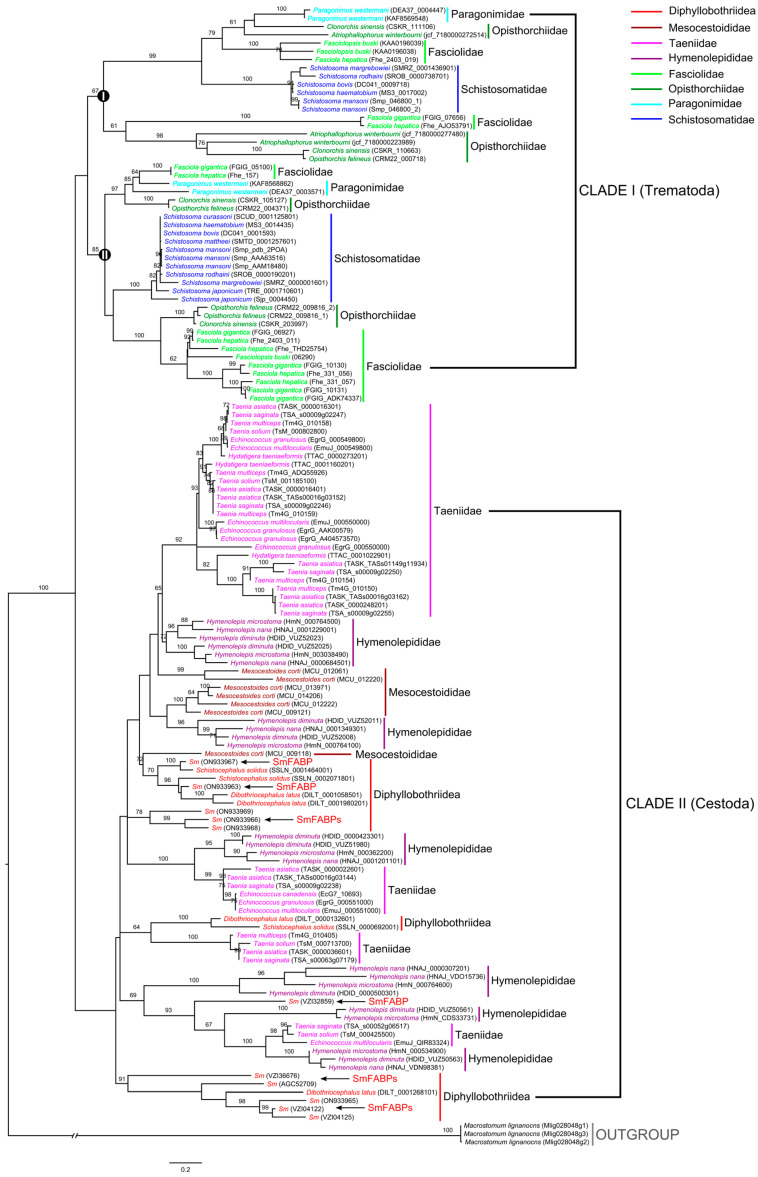
Phylogenetic analysis of fatty-acid-binding protein sequences in medical helminths (cestodes + trematodes) based on the maximum-likelihood method. The FABP sequences from *S. mansoni* are represented by arrows. Values on branches represent bootstrap values, and only values with bootstrap values of >60 are displayed.

A total of 103 FABPs in 15 cestodes and 61 FABPs in 18 trematodes were retrieved from public databases (Supplementary Table S2). The MEME program identified 10 highly conserved specific putative motifs (Supplementary Figure S2). For cestodes, the most frequently appearing motif combination was the motif permutation of 1 + 4 + 2 + 5 + 3 (47%) among a total of 19 motif permutations, followed by motif 1 + 4 + 2 + 5 (10.7%), motif 1 + 6 + 2 + 3 (10.7%), and motif 1 + 4 + 2 + 3 (6.8%). For trematodes, motifs 2 + 5 + 1 + 4 and 2 + 6 + 3 + 1 + 4 appeared most frequently, followed by motifs 2 + 8 + 3 + 1 + 4 (8.2%) and 2 + 6 + 3 + 1 + 4 (6.6%). In the phylogenetic analysis, FABPs were clustered according to their taxonomic systems; the trematodes and cestodes were grouped into a single clade (Clade I and Clade II), although without highly supported values (Figure 2). Within the Trematoda clade, two subclades (subclade I and subclade II) were revealed. Species in four medical fluke families, Opisthorchiidae, Fasciolidae, Paragonimidae, and Schistosomatidae, were dispersed in each generated subclade; nevertheless, trematodes in the same family tended to group together. For the Cestoda clade, although no obvious phylogenetic patterns were observed, species in the same family tended to cluster together as well. The family Hymenolepididae was divided into six separate species groups, showing the most evolutionary diversity in all medical cestodes. Diphyllobothriidea and Taeniidae revealed five and four dispersed groups, respectively. The members of *S. mansoni* were scattered in nearly all the Diphyllobothriidea groups, indicating a high genetic diversity.

### 3.3. Molecular Characterization of SmFABP

**Figure 3:**
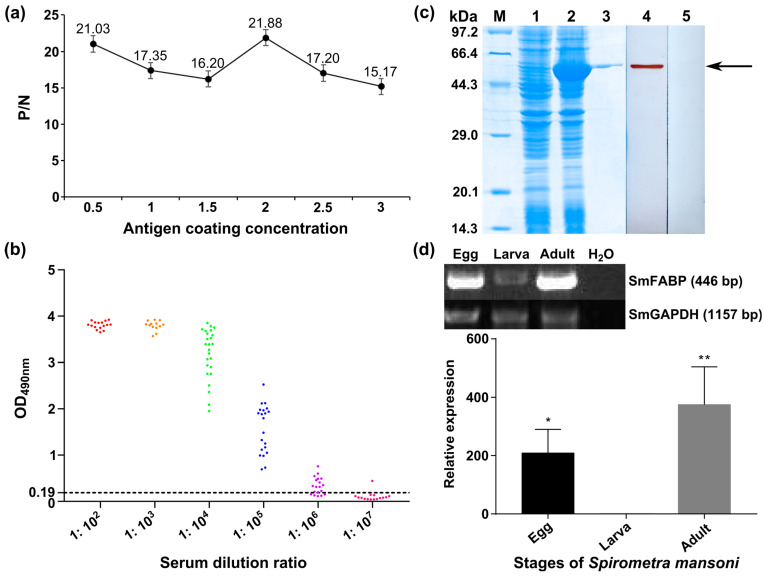
Molecular characterization of SmFABP. (**a**) Determination of the optimal antigen coating concentration. All data are presented as the means and standard deviation (SD) with at least three repeats. (**b**) Determination of anti-rSmFABP immune serum titer by indirect ELISA. The data were obtained from three repeated experiments. Red, orange, green, blue, purple, and rose red represent serum dilutions of 1:10^2^, 1:10^3^, 1:10^4^, 1:10^5^, 1:10^6^, and 1:10^7^, respectively. (**c**) SDS–PAGE and Western blot analyses of the recombinant protein SmFABP. The loading amount of SDS was 5 μg per lane. M: protein marker; Lane 1: uninduced bacterial cultures; Lane 2: induced bacterial cultures; Lane 3: purified rSmFABP; Line 4: rSmFABP + anti-rSmFABP serum; Line 5: rSmFABP + preimmune serum. (**d**) Transcription pattern of the FABP gene in different developmental stages of *Spirometra mansoni*. M: DL2000 Marker; Egg: egg stage; Larva: plerocercoid stage; Adult: adult stage; H_2_O: Milli-Q water control. * represents a statistically significant difference compared with the plerocercoid group (*p* < 0.05), ** represents a statistically significant difference compared with the plerocercoid group (*p* < 0.01). A housekeeping gene (Sm-GAPDH) was used as a positive control. H_2_O was used as a negative control.

**Figure 4:**
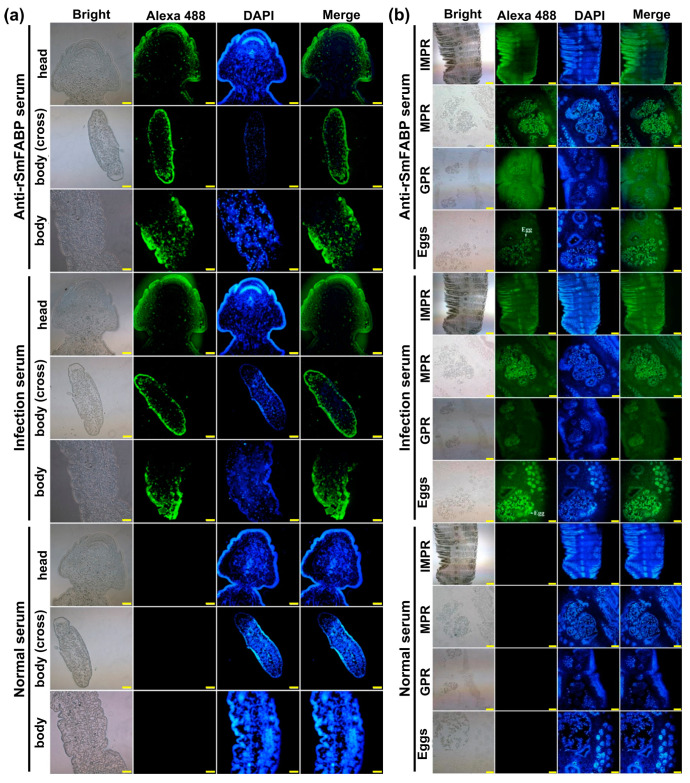
Immunofluorescence localization of FABP in different developmental stages of *S. mansoni*. (**a**) Immunofluorescence localization of SmFABP in the plerocercoid stage. Green fluorescence: SmFABP immune serum/infected mouse serum binding; blue fluorescence: nuclear DAPI staining. Head: the head of plerocercoid; body (cross): transverse section of plerocercoid; body: longitudinal section of plerocercoid; head and body (cross) scale: 200 μm; body scale: 100 μm. (**b**) Immunofluorescence localization of SmFABP in the adult stage. Green fluorescence: SmFABP immune serum/infected cat serum binding; blue fluorescence: DAPI staining of the nuclei. IMPR: immature proglottids; MPR: mature proglottids; GPR: gravid proglottids; IMPR scale: 500 μm; MPR, GPR and eggs scale: 200 μm.

The molecular biological analysis showed that the SmFABP gene encoded 130 amino acids with a molecular weight of 14.49 kDa and an isoelectric point of 5.88. The coding sequence of the SmFABP gene was cloned into the prokaryotic expression vector pMAL-c2X to construct the recombinant expression plasmid. After induction with 0.5 mM IPTG, BL21 bacteria harboring the recombinant plasmid pMAL-c2X-SmFABP expressed a soluble fusion protein. In addition, purified rSmFABP was used to immunize mice to prepare immune serum. An indirect ELISA was established using rSmFABP. A protein concentration of 2.0 μg/mL was the optimal condition (Figure 3a). A cut-off value of 0.21 was used as a standard. The titers of the antisera were above 10^5^ (Figure 3b). In the SDS–PAGE analysis, 5 μg of protein was added to each lane and the molecular size of rSmFABP was 56.5 kDa, consistent with the predicted combined size of the protein encoded by the cDNA clone (14.5 kDa) and maltose-binding protein (MBP) tag from the vector (42 kDa) (Figure 3c). A solubility analysis showed that most rSmFABP was expressed in the supernatant. A Western blotting analysis showed that rSmFABP was recognized by the anti-rSmFABP serum (Figure 3c). mRNA transcription of the SmFABP gene was observed at the egg, plerocercoid, and adult worm stages. A qPCR analysis showed that the transcriptional level of the adult stage was the highest, followed by the egg and plerocercoid stages (Figure 3d). Immunolocalization tests showed that green fluorescence was observed in the head segment of the plerocercoid, the transverse section of the body, and the longitudinal section of the body and was mainly concentrated in the cortex and parenchymal tissue (Figure 4a). In adults, green fluorescence was detected in immature proglottids (IMPR), mature proglottids (MPR), gravid proglottids (GPR), the uterus, and eggs in the uterus. In detail, FABPs were concentrated in the cortex of the worm body, parenchyma, reproductive pores, uterus, and egg shell (Figure 4b).

### 3.4. Fatty Acid Binding Assay

**Figure 5:**
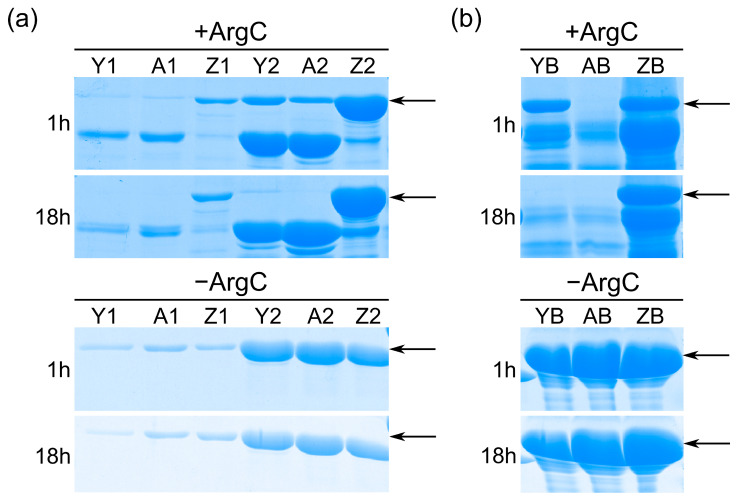
Degradation pattern and stability of recombinant SmFABP and albumin after incubation with ArgC. (**a**): The degradation pattern and stability of rSmFABP with fatty acid. A1, A2: Apo form rSmFABP; Y1, Y2: rSmFABP is mixed with oleic acid; Z1, Z2: rSmFABP is mixed with palmitic acid; +ArgC: protein incubated with ArgC at 37 °C for 1 h and 18 h; −ArgC: protein without ArgC at 37 °C for 1 h and 18 h. (**b**): The degradation pattern and stability of albumin with fatty acid. AB: albumin detected in parallel with recombinant SmFABP; YB: albumin is mixed with oleic acid; ZB: albumin is mixed with palmitic acid. Arrows indicate SmFABP and albumin.

Initially, when incubated with ArgC at 37 °C in the absence fatty acids, the recombinant protein was significantly degraded at 1 h and almost completely degraded at 18 h. When rSmFABP was incubated with oleic or palmitic acid and then incubated with ArgC, it was in a stable state. Moreover, after incubation with palmitic acid, rSmFABP showed little change when incubated with ArgC for either 1 h or 18 h. In comparison, after incubation with oleic acid, rSmFABP was degraded at 18 h. In the negative control, rSmFABP, rSmFABP–oleic acid, and rSmFABP–palmitic acid showed little degradation at either 1 h or 18 h (Figure 5a). Additionally, using albumin as a positive control, SDS–PAGE analysis revealed patterns of proteolysis similar to those of rSmFABP (Figure 5b). 

### 3.5. Fatty Acid Starvation Induction Test

**Figure 6:**
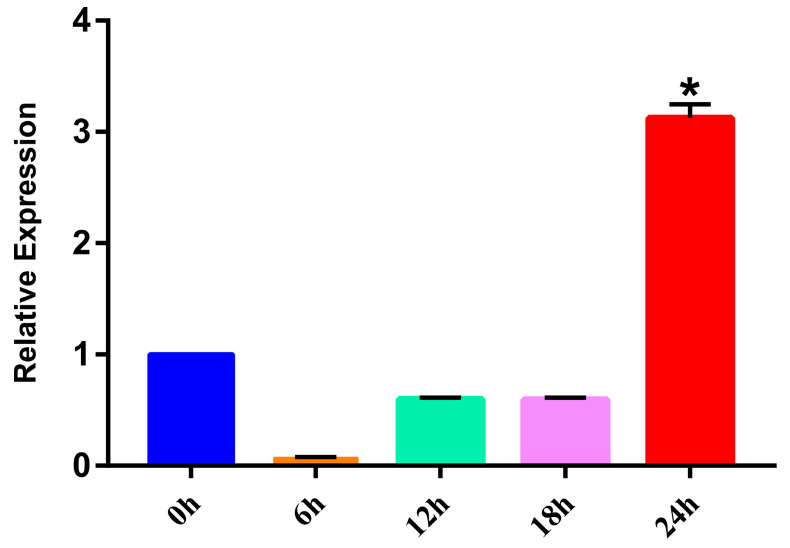
Expression of FABP in vitro culture of plerocercoids. The expression level of FABP in the plerocercoid at 0 h, 6 h, 12 h, 18 h, and 24 h after the fatty acid supply was cut off. The results were generated from the average of three independent replicates. Error bars represent SD (*n* = 3). The expression level of FABP at 0 h was used as a control. * represents a statistically significant difference compared with the expression level of FABP at 0 h (*p* < 0.05).

The expression level of SmFABP at different culture times was observed. Using the 0 h transcriptional level as a control, real-time PCR tests showed that the expression of FABP increased significantly (*p* < 0.05) after culturing for 24 h, suggesting that cutting off the fatty acid supply may promote SmFABP expression (Figure 6). However, at other time points, the expression of FABP was relatively low, possibly because the starvation-induced expression of FABP requires a certain amount of time to accumulate. 

## 4. Discussion

In this study, we reported the identification and characterization of FABPs using all available omic data on *S. mansoni* for the first time. A total of 11 new SmFABPs were identified. In comparison, many different types of FABPs have been identified in other cestodes, including six FABPs in *Echinococcus multilocularis* and *E. granulosus*, two in *Mesocestoides vogae* and *Taenia solium*, one in *T. multiceps*, and one in *T. pisiformis*, indicating high family diversity of FABPs in tapeworms [13,21,41]. Among the 11 SmFABPs, except for SmFABP9 and SmFABP11, all SmFABPs contained a conserved P2 ligand-binding (Arg-x-Tyr) motif involved in the ligand binding of the typical fatty-acid-binding protein as described previously [13]. Interestingly, the SmFABPs with the conserved P2 ligand-binding motif grouped together in the clustering analysis and revealed similar motif organizations in the MEME analysis, indicating the relatively high conservation of SmFABPs. In the 3D structure, the protein contains an intracellular fatty-acid-binding protein domain, consisting of a beta barrel and a spiral-rotating “cap”, which delineates the ligand-binding cavity [42]. An analysis of the expression patterns of SmFABPs displayed ubiquitous but highly variable expressions in all tissues/organs studied. A total of 11 genes were highly expressed in the adult stage, which is in line with the functional features of FABP that contribute to acquiring and utilizing host fatty acids for the parasite [43]. During the life cycle of *S. mansoni*, adults grow rapidly and require more energy and upregulated FABPs can help the worm obtain enough energy to meet its metabolic needs [24].

In the phylogenetic analysis, the FABPs of both cestodes and trematodes tended to group according to their taxonomic classification, suggesting the relative conservation of FABPs in medical platyhelminths, as previously mentioned [21]. FABP genes have only been found in metazoans, and evolutionary studies have distinguished major subfamilies that could have been derived from a single ancestral gene that evolved from a lipocalin gene and underwent the first duplication approximately 930 million years ago [25]. The SmFABPs identified in this study were heterogeneous in regard to length, composition, and identity, possibly driven by the need to transport numerous different fatty acids [29]. Alternative splicing is a major mechanism accounting for the complexity and diversity of FABP protein functions [28]; however, the exact diversification mechanism of SmFABPs is still unknown and needs further study.

To investigate the molecular functions of FABP in *S. mansoni*, a representative SmFABP was successfully expressed and purified in vitro. rSmFABP immunization in BALB/c mice can induce a high specific anti-rSmFABP IgG response. Western blotting revealed that the rSmFABP protein expressed in the cholangiocytes had good antigenicity and high specificity and sensitivity. FABPs often show significant tissue- and time-specific expression. In *E. granulosus*, EgFABP1 is specifically expressed in the larval stage of procercoids, and its expression is closely related to the development of protoscolex larvae [44]. Here, both RT–PCR and qRT–PCR showed that SmFABP was transcribed in eggs, plerocercoids, and adults, indicating important roles of SmFABP in the growth, development, and reproduction of *S. mansoni*. SmFABP was mainly expressed in the egg shell, cortex, and parenchyma of the plerocercoid and in the cortex, parenchyma, uterus, and reproductive pores of adult worms. In addition, positive staining was found in eggs within the uterus of gravid proglottids, suggesting that the energy produced by the oxidation of fatty acids may be necessary for egg development and survival [45].

FABPs are a class of cytoplasmic polygenic proteins that noncovalently bind hydrophobic ligands (mainly fatty acids). These genes are mainly involved in binding/sequestering free fatty acids in the cytosol and the transport of fatty acids (FAs) from the plasma membrane to intracellular transformation sites [46], and several members can participate in the regulation of cell growth and proliferation by mainly binding and transporting fatty acids, which are important mediators in regulating fat storage and distribution [47,48]. Although different FABPs in cestodes have been reported [21,49], few studies have focused on the physiological function of tapeworm FABPs. In this study, SmFABP exhibited typical hydrophobic ligand-binding properties, consistent with FABP members of other species [25,49]. The binding of FABP to fatty acids probably contributed to protecting the hydrolytic sites of FABPs, thus improving their stability in adverse conditions to resist enzymatic degradation. Moreover, we found that rSmFABP appears to form relatively stable binding forms with palmitic acid, although the stability of the conformational change is relatively limited. Different fatty acids have different affinities for FABPs [50,51]; rSmFABP seems to bind more stably to palmitic acid than oleic acid, which is inconsistent with previous studies [52]. Additionally, to explore whether SmFABP participates in lipid-metabolism-related activities, a fatty acid starvation induction test was performed in this study. The results showed that the expression of SmFABP was significantly upregulated after 24 h of in vitro culture. In short, the fatty acid starvation induction test suggested that SmFABP might be involved in fatty acid absorption, transport, and metabolism in the parasite. 

## 5. Conclusions

To elucidate the genes involved in adaptive parasitization of *S. mansoni*, we screened and obtained 11 new SmFABP genes in this study. Their expression patterns at different stages were studied by qPCR. A high genetic diversity of SmFABPs and the relative conservative property of FABPs in medical platyhelminthes were observed in the phylogenetic analysis. In addition, the molecular characteristics of SmFABP were investigated. SmFABP is mainly located in the egg shell, tegument, and parenchymal tissue of the plerocercoid, uterus, genital pores, and cortex of the adult worm. The fatty acid binding assay showed that when binding with palmitic acid, rSmFABP can build a more stable holo form to protect the hydrolytic sites of FABPs. The fatty acid starvation induction test suggested that SmFABP might be involved in fatty acid absorption, transport, and metabolism in *S. mansoni*.

## Figures and Tables

**Table 1:** Annotations features of the fatty-acid-binding protein of *Spirometra mansoni*.

Gene ID	CDS Length (bp)	Protein (aa)	FABP Domain Coordinates	Domain Length (aa)	Mw (Da)	PI	Subcellular Location
ON933963	462	153	5–152	148	17,481.98	8.09	other
ON933964	393	130	3–129	127	14,491.84	5.88	other
ON933965	399	132	3–131	129	14,686.97	6.72	other
ON933966	393	130	3–129	127	14,610.59	5.61	other
ON933967	417	138	11–137	127	15,372.68	6.11	S
ON933968	393	130	3–129	127	14,792.88	6.91	other
ON933969	396	131	3–130	128	14,544.46	5.42	other
OP146602	225	75	7–75	69	8426.57	5.00	other
OP146603	360	119	1–118	118	13,231.03	6.35	other
OP146604	264	87	3–88	86	10,278.89	6.28	other
OP146605	360	119	3–115	113	13,414.25	6.74	other

## Data Availability

The data supporting the conclusions of this article are included within the article.

## Supplementary Materials

The following supporting information can be downloaded at: https://www.mdpi.com/article/10.3390/ani13182855/s1, Figure S1: 3D analysis of SmFABP. (a) Tertiary structure prediction of SmFABP; (b) Quality assessment of tertiary structural models; Figure S2: Conserved motifs of FABP sequence motifs in cestodes and trematodes; Table S1: Primer sets used in qRT–PCR analysis; Table S2: Summary of fatty acid binding proteins in cestodes and trematodes.
