# Supplementary material for: Molecular Characteristics of the Fatty-Acid-Binding Protein (FABP) Family in Spirometra mansoni―A Neglected Medical Tapeworm
Source: Animals (Basel). 2023 Sep 8;13(18):2855. doi: 10.3390/ani13182855 (PMC10525997; doi:10.3390/ani13182855)
Supplement: Supplementary file 1 [file animals-13-02855-s001.zip › animals-2578034-supplementary.pdf]

Supplementary materials

Molecular Characteristics of the Fatty-Acid-Binding Protein (FABP) Family in *Spirometra mansoni*—A Neglected Medical Tapeworm

Table S1. Primer sets used in qRT–PCR analysis.

Table S2. Summary of fatty acid binding proteins in cestodes and trematodes.

Figure S1. 3D analysis of SmFABP. (a) Tertiary structure prediction of SmFABP; (b) Quality assessment of tertiary structural models.

Figure S2. Conserved motifs of FABP sequence motifs in cestodes and trematodes.

Table S1. Primers used in qRT-PCR analysis.

Gene ID	Primer name	Sequence (5'-3')	Product size (bp)
ON933963	ON933963-F<br>ON933963-R	CTGACAATCACGAAGGAGGG AGTG-<br>GACTTTACTTTGCGACC	132
ON933964	ON933964-F<br>ON933964-R	GCCTGTTGGCGATGGTTA AC-<br>CTGTTTCAGCGTCTTGC	153
ON933965	ON933965-F<br>ON933965-R	GATGACTATTACATTGGAGGGC<br>AACTTTGACGTAGTGTCGAACA	148
ON933966	ON933966-F<br>ON933966-R	AGCGTTTGGGTTTTAATCTG<br>ATCCATCGTGGTCAAGTCG	167
ON933967	ON933967-F<br>ON933967-R	ATTATGGATGCCTTCCTTGG<br>CGTCGGTTTCATTGCGTTA	120
ON933968	ON933968-F<br>ON933968-R	AGAGCGCCTTGGCTTCAG<br>CGTGTCTTCCATCCATCGT	179
ON933969	ON933969-F<br>ON933969-R	CACCCCACAGTGACCATCG<br>CGTCGCTTTCAACGCATCT	177
OP146602	OP146602-F<br>OP146602-R	GGGCGATGGCTACAGTCT TCGCGTTCAAC-<br>GTAGGTTAC	183
OP146603	OP146603-F<br>OP146603-R	ACTGCCGCAAACACCCTG CGCCTTCAAC-<br>GCATCTCC	192
OP146604	OP146604-F<br>OP146604-R	CTTTGTTGAGTACCTGCGTTGT CTTTGTTGAG-<br>TACCTGCGTTGT	94
OP146605	OP146605-F<br>OP146605-R	CTGACAATCACGAAGGAGGG AGTG-<br>GACTTTACTTTGCGACC	132
GAPDH	GAPDH-F<br>GAPDH-R	AGCAACCTCGTTGATGTCGT TGAATTGAC-<br>CGTGGGTGGAG	97

Table S2. Summary of fatty acid binding proteins in cestodes and trematodes.

Class	Family	Genus	Species	SS	Sequence ID（Gene）
Cestoda	Diphyllobothriidea	*Dibothriocephalus*	*D. latus*	4	DILT_0000132601, DILT_0001268101, DILT_0001058501, DILT_0001980201
		Spirometra	*S. mansoni*	11	Sm_AGC52709, Sm_ON933963, Sm_ON933965, Sm_ON933966, Sm_ON933967, Sm_ON933968, Sm_ON933969, Sm_VZI04125, Sm_VZI04122, Sm_VZI32859, Sm_VZI36676

	Schistocephalus	*S. solidus*	3	SSLN_0001728001, SSLN_0001464001, SSLN_0000692001
Taeniidae	*Echinococcus*	*E. canadensis*	1	EcG7_10693
		E. granulosus	5	EgrG_000549800, EgrG_000551000, EgrG_000550000, EgrG_AAK00579, EgrG_A404573570,
		E. multilocularis	4	EmuJ_000550000, EmuJ_000549800, EmuJ_000551000, EmuJ_QIR83324
	Taenia	*T. asiatica*	9	TASK_0000022601, TASK_0000016301, TASK_0000016401, TASK_0000248201, TASK_0000036601, TASK_TASs00016g03152, TASK_TASs00016g03144, TASK_TASs01149g11934, TASK_TASs00016g03162
		T. saginata	7	TSA_s00009g02238, TSA_s00063g07179, TSA_s00009g02246, TSA_s00009g02247, TSAs_00009g02255, TSA_s00009g02250, TSA_s00052g06517
		T. solium	4	TsM_001185100, TsM_000802800, TsM_000713700, TsM_000425500
		T. multiceps	6	Tm4G_010159, Tm4G_010154, Tm4G_010158, Tm4G_010150, Tm4G_010405, Tm4G_ADQ55926
	Hydatigera	*H. taeniaeformis*	3	TTAC_0000273201, TTAC_0001160201, TTAC_0001022901
Hymenolepididae	*Hymenolepis*	*H. diminuta*	9	HDID_0000423301, HDID_0000500301, HDID_VUZ50563, HDID_VUZ50561, HDID_VUZ52025, HDID_VUZ52023, HDID_VUZ52011, HDID_VUZ52008, HDID_VUZ51980
		H. microstoma	7	HmN_000362200, HmN_000764500, HmN_000764600, HmN_000534900, HmN_000764100, HmN_003038490, HmN_CDS33731
		H. nana	7	HNAJ_0001229001, HNAJ_0001201101, HNAJ_0000307201, HNAJ_0001349301, HNAJ_0000684501, HNAJ_VDN98381, HNAJ_VDO15736

	Mesocestoididae	*Mesocestoides*	*M. corti*	7	MCU_012220, MCU_014206, MCU_009118, MCU_012061, MCU_012222, MCU_013971, MCU_009121,
		Atriophallophorus	*A. winterbourni*	3	jcf_7180000277480, jcf_7180000272514, jcf_7180000223989
	Opisthorchiidae	*Clonorchis*	*C. sinensis*	4	CSKR_110663, CSKR_105127, CSKR_111106, CSKR_203997
		Opisthorchis	*O. felineus*	4	CRM22_000718, CRM22_004371, CRM22_009816.1, CRM22_009816.2
		Fasciolopsis	*F. buski*	3	FBUS_06290, FBUS_KAA0196039, FBUS_KAA0196038
	Fasciolodae	*Fasciola*	*F. gigantica*	6	FGIG_06927, FGIG_10130, FGIG_10131, FGIG_05100, FGIG_07656, FGIG_ADK74337
Trematoda			*F. hepatica*	7	Fhe_157, Fhe_2403_0.19, Fhe_331_0.57, Fhe_2403_0.11, Fhe_331_0.56, Fhe_THD25754, Fhe_AJO53791
	Paragonimidae	*Paragonimus*	*P. westermani*	4	DEA37_0003571, DEA37_0004447, DEA37_KAF8569548, DEA37_KAF8568862
			S. bovis	2	DC041_0001593, DC041_0009718
			S. curassoni	1	SCUD_0001125801
			S. haematobium	2	MS3_0017002, MS3_0014435
			S. japonicum	2	Sjp_0004450, Sjp_TRE_0001710601
	Schistosomatidae	*Schistosoma*	*S. mansoni*	5	Smp_046800.1, Smp_046800.2, Smp_P29498, Smp_AAM18480, Smp_pdb|2POA
			S. margrebowiei	2	SMRZ_0001436901, SMRZ_0000001601
			S. mattheei	1	SMTD_0001257601
			S. rodhaini	2	SROB_0000190201, SROB_0000738701

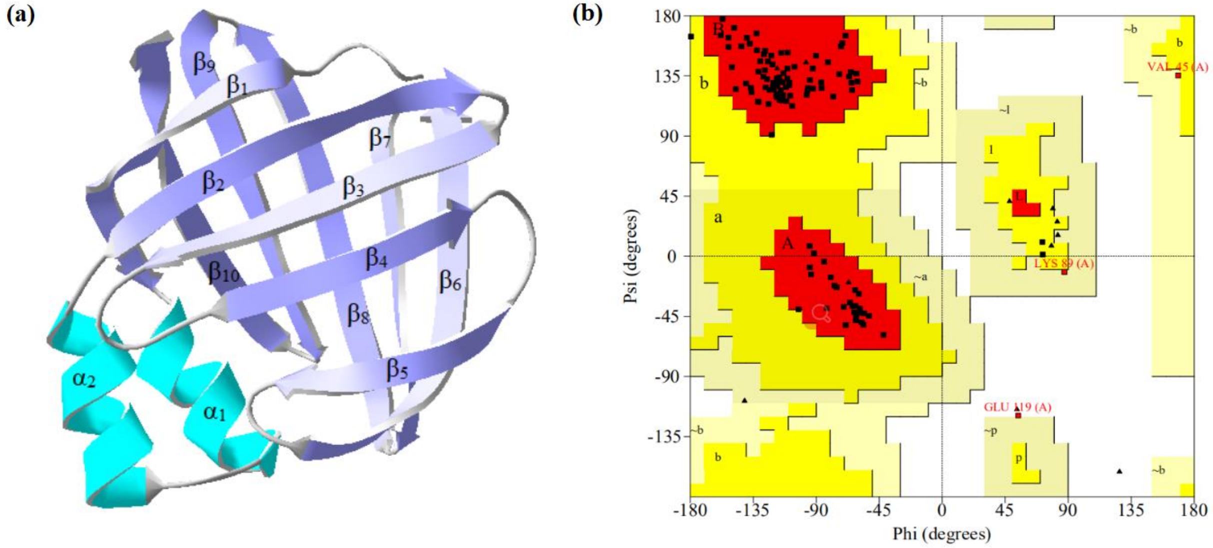

Figure S1. 3D analysis of SmFABP. (a) Tertiary structure prediction of SmFABP; (b) Quality assessment of tertiary structural models.

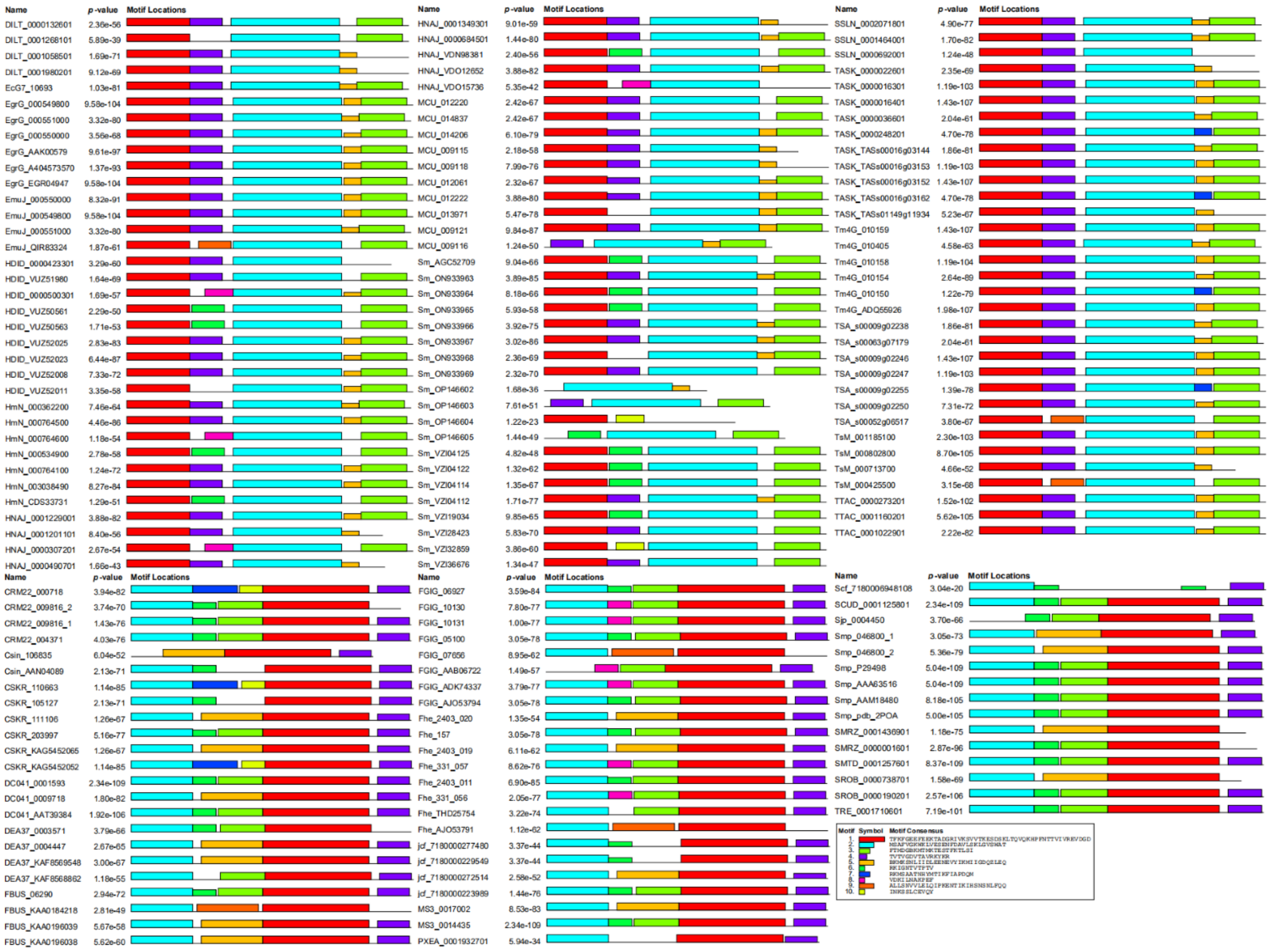

Figure S2. Conserved motifs of FABP sequence motifs in cestodes and trematodes.
